# Reply to “Subgenome-aware analyses suggest a reticulate allopolyploidization origin in three *Papaver* genomes”

**DOI:** 10.1038/s41467-023-37940-9

**Published:** 2023-04-19

**Authors:** Xiaofei Yang, Shenghan Gao, Tun Xu, Bo Wang, Yanyan Jia, Kai Ye

**Affiliations:** 1 Genome Institute, the First Affiliated Hospital of Xi’an Jiaotong University, Xi’an, 710049 Shaanxi China; 2 School of Computer Science and Technology, Faculty of Electronic and Information Engineering, Xi’an Jiaotong University, Xi’an, 710049 Shaanxi China; 3 MOE Key Lab for Intelligent Networks & Networks Security, Faculty of Electronic and Information Engineering, Xi’an Jiaotong University, Xi’an, 710049 Shaanxi China; 4 School of Automation Science and Engineering, Faculty of Electronic and Information Engineering, Xi’an Jiaotong University, Xi’an, 710049 Shaanxi China; 5 School of Life Science and Technology, Xi’an Jiaotong University, Xi’an, 710049 Shaanxi China; 6 Faculty of Science, Leiden University, 2311 EZ Leiden, The Netherlands

**Keywords:** Plant evolution, Evolutionary genetics, Adaptive radiation, Genome evolution

**replying to** R.-G. Zhang *et al. Nature Communications* 10.1038/s41467-023-37939-2 (2023)

We assembled genomes of three *Papaver* species, *P. somniferum, P. rhoeas* and *P. setigerum*, and revealed two-round whole genome duplication (WGD) events and the punctuated patchwork evolution of morphinan and noscapine biosynthesis pathway based on comparative genomic analyses of collinearity, phylogenetic tree, and synonymous substitution rate (*Ks*) at species level^1^. Recently, Zhang et al. reanalyzed our three *Papaver* genomes based on the subgenome-phasing analysis by their newly published tool SubPhaser^2^. They confirmed the two WGDs we previously detected, but provide strong evidence that the WGDs were likely allopolyploidy events. We consider the subgenome-phasing analysis from Zhang et al. to be an important research development building on our original study^1^, promoting the illuminating the evolutionary history of *Papaver* species.

Although alternative model proposed by Zhang et al. based on subgenome-phasing analysis further the complicated evolutionary history of *Papaver* species, the actual types of WGDs and the true evolution history of *Papaver* species are still open questions for further investigation. Both novel computational methods and high-quality genomes of more species, especially those closely related species for the subgenomes A, B, C, and D, are required and welcome to further advance our interpretation of the evolutionary model of *Papaver* species, key gene clusters and key genes, like *STORR*.

## Reporting Summary

Further information on research design is available in the Nature Portfolio Reporting Summary linked to this article.

## Supplementary information

Reporting Summary

## Data Availability

Data sharing not applicable to this article as no new datasets were generated during the current study.
